# Supplementary figures and images for: Effect of geometric distortion correction on thickness and volume measurements of cortical parcellations in 3D T1w gradient echo sequences
Source: PLoS One. 2023 Apr 14;18(4):e0284440. doi: 10.1371/journal.pone.0284440 (PMC10104308; doi:10.1371/journal.pone.0284440)

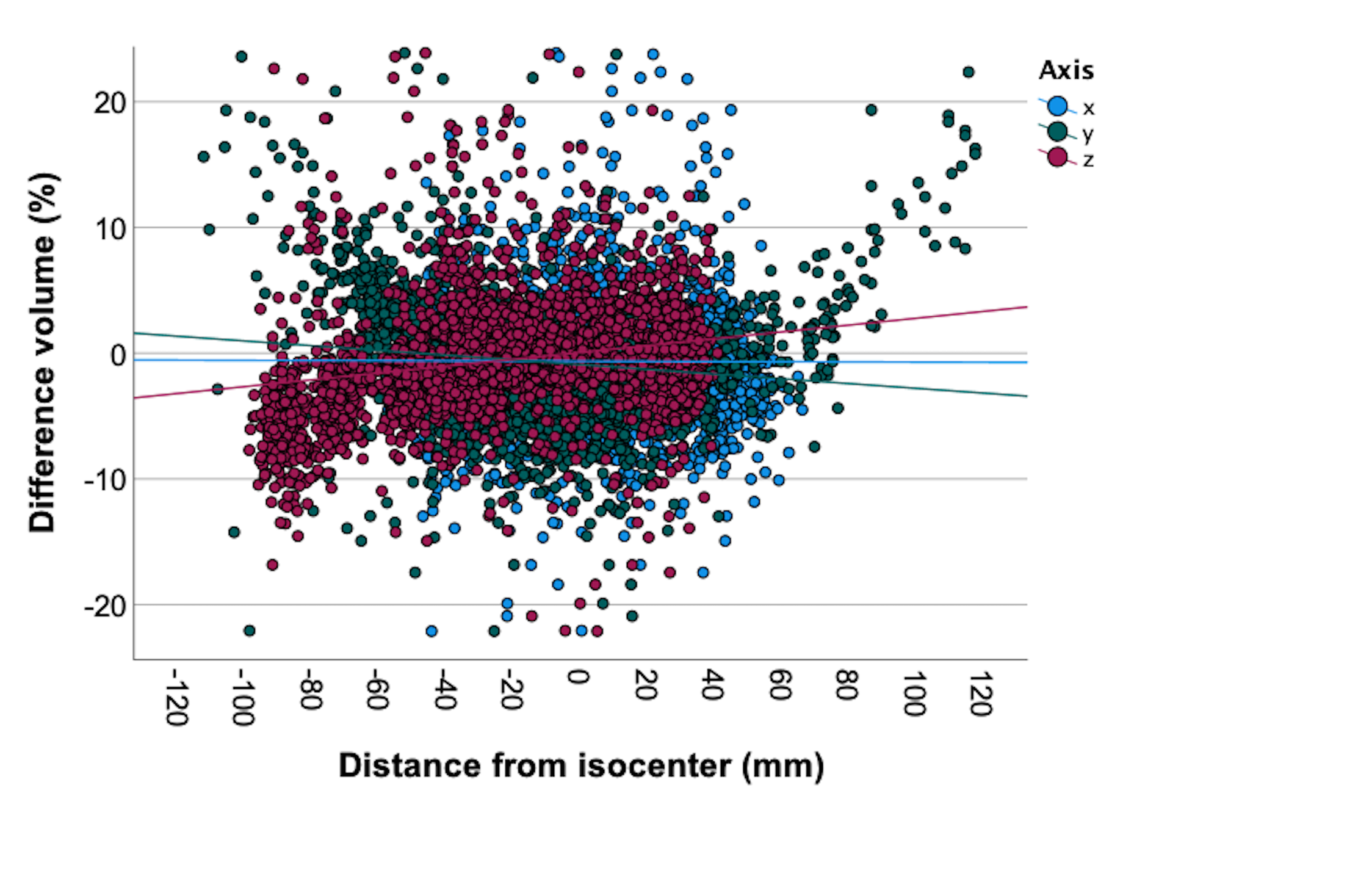

Supplement: S1 Fig — Values are displayed for each axis (x,y,z) in separate colors. (TIF) [file pone.0284440.s001.tif]

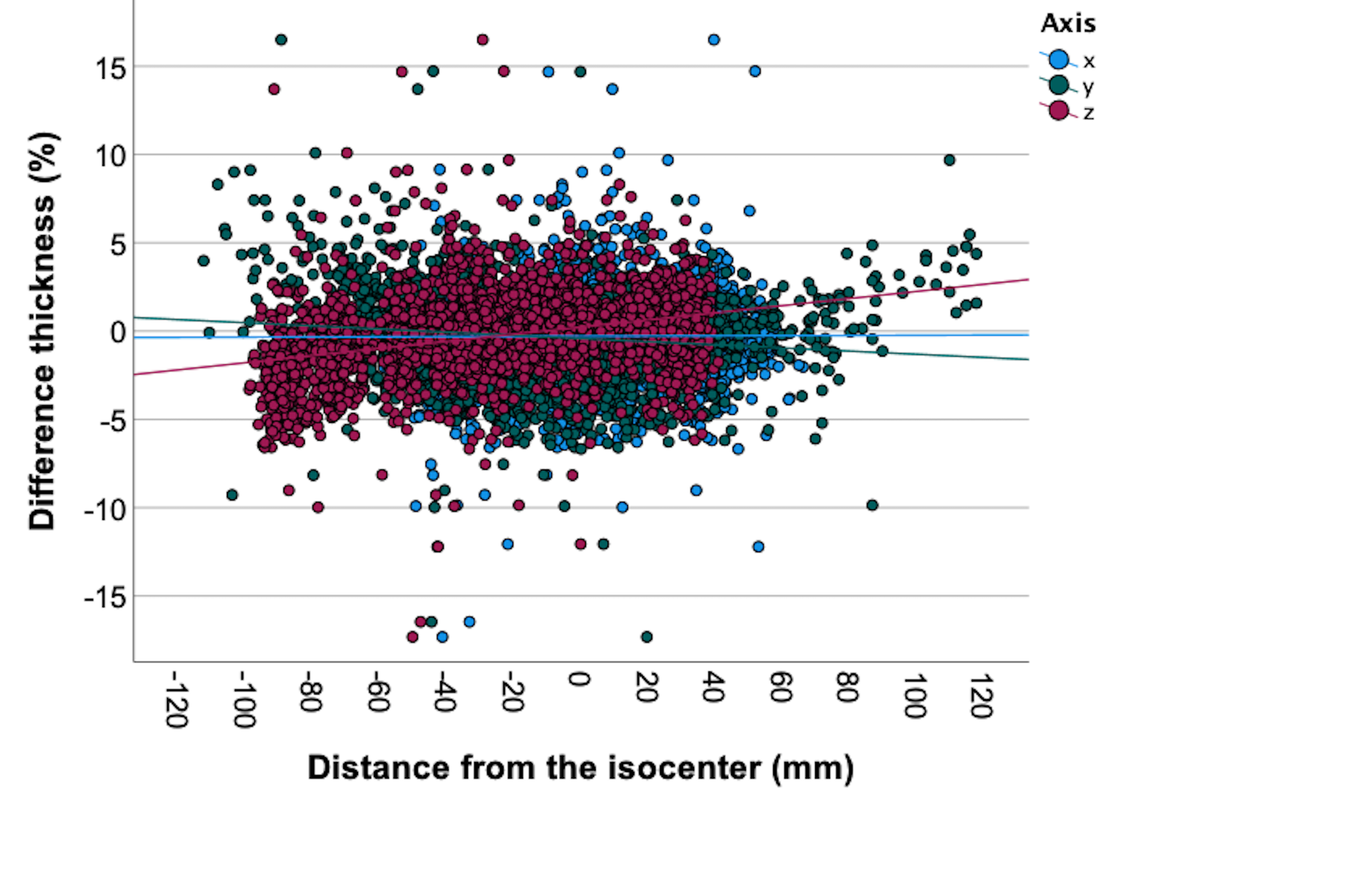

Supplement: S2 Fig — Values are displayed for each axis (x,y,z) in separate colors. (TIF) [file pone.0284440.s002.tif]
