# Supplementary material for: Effect of geometric distortion correction on thickness and volume measurements of cortical parcellations in 3D T1w gradient echo sequences
Source: PLoS One. 2023 Apr 14;18(4):e0284440. doi: 10.1371/journal.pone.0284440 (PMC10104308; doi:10.1371/journal.pone.0284440)
Supplement: S1 Table — (DOCX) [file pone.0284440.s003.docx]

|  | CCC thickness | lower CI | upper CI | CCC volume | lower CI | upper CI |
| --- | --- | --- | --- | --- | --- | --- |
| caudal anterior cingulate | 0.939 | 0.904 | 0.961 | 0.956 | 0.931 | 0.972 |
| caudal middle frontal | 0.925 | 0.887 | 0.951 | 0.665 | 0.524 | 0.770 |
| cuneus | 0.863 | 0.793 | 0.910 | 0.934 | 0.898 | 0.957 |
| entorhinal | 0.912 | 0.863 | 0.944 | 0.927 | 0.887 | 0.953 |
| fusiform | 0.938 | 0.904 | 0.961 | 0.954 | 0.927 | 0.971 |
| inferior parietal | 0.877 | 0.822 | 0.916 | 0.934 | 0.901 | 0.957 |
| inferior temporal | 0.958 | 0.934 | 0.973 | 0.957 | 0.933 | 0.973 |
| insula | 0.933 | 0.895 | 0.957 | 0.976 | 0.962 | 0.985 |
| isthmus cingulate | 0.919 | 0.875 | 0.948 | 0.926 | 0.886 | 0.953 |
| lateral occipital | 0.783 | 0.700 | 0.845 | 0.916 | 0.875 | 0.944 |
| lateral orbitofrontal | 0.942 | 0.909 | 0.963 | 0.973 | 0.957 | 0.983 |
| lingual | 0.873 | 0.805 | 0.918 | 0.943 | 0.910 | 0.964 |
| medial orbitofrontal | 0.910 | 0.863 | 0.942 | 0.972 | 0.955 | 0.982 |
| middle temporal | 0.960 | 0.937 | 0.974 | 0.974 | 0.961 | 0.983 |
| paracentral | 0.855 | 0.781 | 0.905 | 0.776 | 0.675 | 0.848 |
| parahippocampal | 0.969 | 0.951 | 0.980 | 0.907 | 0.856 | 0.941 |
| pars opercularis | 0.949 | 0.920 | 0.968 | 0.933 | 0.895 | 0.957 |
| pars orbitalis | 0.950 | 0.921 | 0.968 | 0.962 | 0.940 | 0.976 |
| pars triangularis | 0.951 | 0.923 | 0.969 | 0.935 | 0.899 | 0.958 |
| pericalcarine | 0.914 | 0.868 | 0.944 | 0.953 | 0.928 | 0.969 |
| postcentral | 0.470 | 0.293 | 0.615 | 0.946 | 0.917 | 0.965 |
| posterior cingulate | 0.870 | 0.802 | 0.916 | 0.920 | 0.876 | 0.948 |
| precentral | 0.577 | 0.427 | 0.696 | 0.801 | 0.708 | 0.867 |
| precuneus | 0.905 | 0.853 | 0.939 | 0.843 | 0.763 | 0.897 |
| rostral anterior cingulate | 0.933 | 0.895 | 0.957 | 0.988 | 0.981 | 0.993 |
| rostral middle frontal | 0.954 | 0.927 | 0.971 | 0.935 | 0.899 | 0.959 |
| superior frontal | 0.889 | 0.834 | 0.926 | 0.919 | 0.876 | 0.947 |
| superior parietal | 0.914 | 0.870 | 0.943 | 0.733 | 0.611 | 0.820 |
| superior temporal | 0.969 | 0.952 | 0.981 | 0.972 | 0.956 | 0.982 |
| supramarginal | 0.929 | 0.890 | 0.955 | 0.778 | 0.669 | 0.854 |
| transverse temporal | 0.953 | 0.926 | 0.970 | 0.972 | 0.955 | 0.982 |
