# Supplementary material for: Effect of geometric distortion correction on thickness and volume measurements of cortical parcellations in 3D T1w gradient echo sequences
Source: PLoS One. 2023 Apr 14;18(4):e0284440. doi: 10.1371/journal.pone.0284440 (PMC10104308; doi:10.1371/journal.pone.0284440)
Supplement: Attachment — Submitted filename: Response_to_reviewers.docx [file pone.0284440.s005.docx]

Review Comments to the Author

Please use the space provided to explain your answers to the questions above. You may also include additional comments for the author, including concerns about dual publication, research ethics, or publication ethics. (Please upload your review as an attachment if it exceeds 20,000 characters)

Reviewer #3: This paper investigates the effects of distortion correction on thickness and volume measurements from 3D T1w MR images using FreeSurfer and a commercially available distortion correction algorithm. Adequate data were acquired and processed to support the objective of this study. Careful proofreading, more elaboration, and better presentation of the results are needed.


***Introduction
R1. In the sentence “…scanner system upgrades or movement”, what movement? Scanner movement?

A1. *Thank you for pointing this out. Of course, patient movement was meant, and we corrected the sentence accordingly.*

*“Numerous technical factors can influence the results of brain volumetric analysis, such as acquisition protocols, intra- and interscanner variablilty, scanner system upgrades or patient movement [2, 3].”*

***Materials and Methods
R2. "No additional motion correction was applied." --> "No motion correction was applied."

A2. *The sentence was rephrased accordingly.*

R3. More details about the distortion correction algorithm are needed. Siemens provides two options (2D or 3D) for distortion correction. Which one was used? A brief description of the correction algorithm, which can be obtained from Siemens scientists/staff, will be helpful for readers to be aware of the differences between readers’ algorithms and the algorithm in this work.

A3. *In this study we used a 3D distortion correction. It is difficult to provide specific information about the algorithm since Siemens keeps its software confidential. However, we are able to refer to the patent of the distortion correction algorithm. (https://patents.google.com/patent/DE19540837A1/en)*

R4. Were any filters used?

A4. *No filters were applied to the acquired images.*

*FreeSurfer cortical thickness and volume
R5. Figure 1: It is very hard to visualize blue and purple. While it is good to inspect the whole brain, having close-up or zoomed-in views is needed to appreciate the differences between DC and nDC. For example, see figure 4 in <https://pubmed.ncbi.nlm.nih.gov/31666530/>. Showing the ROIs with most pronounced differences in thickness and volume measurements would be helpful. For example, precentral and paracentral ROIs.

A5. *We fully agree with the reviewer’s comment. It is difficult to visualize the subtle differences caused by distortion correction. For a better visualization, we added a new figure to the manuscript to display the effect of distortion correction. In this new figure, we also zoomed into the central region, where distortion effects were prominent. However, due to the subtle differences it is still difficult to visualize the effect on cortical thickness and volume.*

R6. Use either isocenter or isocentre.

A6. *Thank you for pointing this out. It was changed to isocenter.*

R7. It seems that the last paragraph in this section is to explain how the distance was calculated. In sentence “Isocenter coordinates for each patient were derived from the origin of the anatomical coordinate system that were transferred to the respective image coordinate system”, what is the isocenter in this sentence? Is it the magnetic isocenter mentioned in Statistics?
In “The isocentre … then used to calculate the distance for each FreeSurfer parcel from the isocenter. “, what are the two isocenters? Were they the magnetic isocenter that was mentioned later. Please clarify.
How was the distance calculated?

A7. *The reviewer’s assumption is correct. Every time the isocenter is mentioned, we refer to the magnetic isocenter. The coordinates of the magnetic isocenter can be obtained from the origin of the anatomical coordinate system. However, by using the recon-all command the cortical parcellation will be created in a new “FreeSurfer” space and not in the original T1 space and therefore it is necessary relocate the magnetic isocenter in the FreeSurfer space. Further, we calculated the distance between the magnetic isocenter, now transformed in the FreeSurfer space, and the center of gravity of each ROI.*

*For a better understanding we rephrased the paragraph and added additional information about the determination of the magnetic isocenter in the FreeSurfer space.*

***Results
*Cortical thickness and volume
R8. Since Table 1 is a busy table, adding horizontal gridlines will improve its readability. Further, it may be helpful to add a column on the left to assign a number to each ROI/row. Then use the numbers as y labels in Figures 2 and 3, whose y labels in the current format are too busy/blurry to read. Doing so will greatly improve the visualization of the figures. More comments about Figs 2 and 3 below.

A8. *Thank you very much for this excellent advice. We gladly followed the reviewer’s suggestion by adding horizontal gridline to the table. Also, we added a column with number from 1-31 assigned to the ROI labels.*

*Also, we added those labels to Figs 2 and 3 to improve the visualization.*

R9. In the title of Table 1:
The sentence “A p-value <0.05 was considered statistically significant” appeared in Statistics already and thus is redundant. It may be better to revise the last three sentences as two: “Significant differences (p < 0.05) are highlighted in bold. The false discovery rate (FDR) was used to correct for multiple comparisons.”

*A9. We changed the sentence accordingly.*

R10. In Figures 2 and 3, y labels are too blurry to read. The information of the two figures is similar to/derived from Table 1. While it is nice to visualize the ranking of ROIs in terms of differences, it is more relevant to visualize the dependence of difference on distance, which is directly related to geometric distortion. Therefore, I suggest to add the following figures: mean-percentage-difference vs distance plots for thickness and volume measurements. Include 3 curves in each figure: mean-percentage-difference vs x, y, and z.

*A10. Thank you very much for this comment. We added two new figures to the manuscript as suggested by the reviewer.*

R11. There is a formula/equation in the caption of Figures 2 and 3. It can be moved to be and described better in the section “FreeSurfer cortical thickness and volume”.

*A11. We moved the equation to the section “FreeSurfer cortical thickness and volume”*

R12. In Figure 4, use “Concordance correlation coefficient” is more descriptive/explicit than “CCC”. X labels are blurry.

*A12. We changed the heading of the figure and improved the image’s resolution.*

***Discussion
R13. “… were not included in or study” --> “… were not included in our study”

R14. “For the mixed linear model” --> “For the linear mixed model”

*A13 and A14. Thank you for pointing out these typos. We corrected them in the manuscript accordingly.*

R15. To calculate p values for linear mixed model, please check “Evaluating significance in linear mixed-effects models in R” (<https://link.springer.com/article/10.3758/s13428-016-0809-y>).

A15. *We used the function lmer in R to create the model and the model was used to investigate factors that could influence the difference of the estimates. The p values were determined by using ANOVA. However, we understand that determining p values can be challenging for linear mixed models and it is often recommended to omit them. We added this to the limitations of this paper.*
