# Supplementary material for: Effect of geometric distortion correction on thickness and volume measurements of cortical parcellations in 3D T1w gradient echo sequences
Source: PLoS One. 2023 Apr 14;18(4):e0284440. doi: 10.1371/journal.pone.0284440 (PMC10104308; doi:10.1371/journal.pone.0284440)
Supplement: Attachment — Submitted filename: Response_to_reviewer.docx [file pone.0284440.s006.docx]

Review Comments to the Author

Please use the space provided to explain your answers to the questions above. You may also include additional comments for the author, including concerns about dual publication, research ethics, or publication ethics. (Please upload your review as an attachment if it exceeds 20,000 characters)

Reviewer #3:

R1. acquired within 5m55s -> acquired in 5m55s

A1. We changed the sentence accordingly.

R2. As mentioned previously, please specify 2D or 3D correction used for the study. And discuss/mention possible differences between the 2D and 3D corrections or lack of comparing the two may be a limitation of this study in discussion/limitations.

A2. We added the information to the manuscript that the distortion correction used in this study is a 3D correction. We agree with the reviewer that we cannot compare our results with 2D correction methods which are still used for distortion corrections. To further address this we added following sentences and reference to our limitations.

“Also, we used a 3D correction method and cannot our results with studies using a 2D correction method which are still in use by many vendors. However, the advantage of 3D distortion correction methods is their ability to provide more accurate and precise correction of image distortions, leading to improved image quality, better diagnostic accuracy.[1]”

R3. It is nice to compare images with and without geometry correction in Figs 1A-D.
In Figs 1C and D, if possible please add arrows to point out the regions/edges that show the effects of the correction.
Labels E and F in Fig 1 are missing.
As pointed out by the statement "The distortion effects were most prominent in the central region but difficult to detect visually" in Fig 1 caption, is it possible to have close-up views of an off-isocenter region of E and F to show purple and blue regions better?

A3. We understand the reviewer’s suggestions and tried to display the differences caused by the distortions on 3D T1w images as clearly as possible. Due to the subtle differences it is difficult to clearly demonstrate the distortions in the images. However, we believe that Fig 1 and 2 display the distortion effects in T1w images as accurate as possible. We added the labels E and F in Fig 1.

R4.For Fig 4, my previous suggestion of using label IDs does not seem to be much helpful. In order to improve the readability of this figure, please consider to move the ID/label to close to left and right alternately of the error bars. Doing so, a larger font size can be used. e.g. following is a cartoon version of the aforementioned arrangement:

4.Entorhinal |---------O---------|
|-------------------------O-----------------------------| 8.Insula

And use "Cortical ROIs" as the label for y axis.

A4. Thank you for this suggestion. We changed Figure 3 and 4 accordingly.

R5. In Fig 5, all labels should be in black, instead of gray. These labels are a bit blurry. Font size of X labels need to be increased to be readable.

A5. We changed the colour and font size of the labels.


R6. Supplemental Fig 1 and 2 show that percentage differences in cortical thickness and volume are minimal at a location ~(125,125,125) mm away from the scanner isocenter. This is counter-intuitive. At isocenter, gradient is 0 and B0 is more uniform, shouldn't geometric distortion be less influential? Please clarify.

A6.

We agree with the reviewer that this is counter-intuitive and also not correct. When creating Supplemental Fig 1 and 2 we picked the wrong column for distance from the isocenter. We recreated both figures with the correct data. Now, minimal differences are seen in proximity to the magnetic isocenter.
